# Metabolite concentrations and the expression profiles of the corresponding metabolic pathway genes in eggplant (Solanum melongena L.) fruits of contrasting colors

**DOI:** 10.18699/vjgb-24-69

**Published:** 2024-10

**Authors:** M.A. Filyushin, E.A. Dzhos, A.V. Shchennikova, E.Z. Kochieva

**Affiliations:** Federal Research Centre “Fundamentals of Biotechnology” of the Russian Academy of Sciences, Moscow, Russia; Federal Research Centre “Fundamentals of Biotechnology” of the Russian Academy of Sciences, Moscow, Russia Federal Scientific Vegetable Center, VNIISSOK village, Moscow region, Russia; Federal Research Centre “Fundamentals of Biotechnology” of the Russian Academy of Sciences, Moscow, Russia; Federal Research Centre “Fundamentals of Biotechnology” of the Russian Academy of Sciences, Moscow, Russia

**Keywords:** eggplant cultivars, Solanum melongena L., carotenoids; anthocyanins, soluble sugars, expression of metabolic pathway genes, сорта баклажана, Solanum melongena L., каротиноиды, антоцианы, растворимые сахара, экспрессия генов метаболических путей

## Abstract

Eggplant (Solanum melongena L.) ranks fifth in importance among vegetable crops of the Solanaceae family, in part due to the high antioxidant properties and polyphenol content of the fruit. Along with the popular purple-fruited varieties of S. melongena, there are cultivars, the fruits of which are rich in phenolic compounds, but are white-colored due to the lack of anthocyanin biosynthesis. Determination of the amount of anthocyanins and other phenolic compounds, as well as carotenoids and sugars, is included in the assessment of the quality of eggplant fruits of commercial (technical) ripeness. In addition to antioxidant and taste properties, these metabolites are associated with fruit resistance to various stress factors. In this study, a comparative analysis of the content of anthocyanins, carotenoids and soluble sugars (sucrose, glucose, fructose) in the peel and pulp of the fruit of both technical and biological ripeness was carried out in purple-fruited (cv. Vlas) and white-fruited (cv. Snezhny) eggplant accessions of domestic selection. The peel and pulp of biologically ripe fruits of the cvs Vlas and Snezhny were used for comparative transcriptomic analysis. The key genes of the flavonoid and carotenoid metabolism, sucrose hydrolysis, and soluble sugar transport were shown to be differentially expressed between fruit tissues, both within each cultivar and between them. It has been confirmed that the purple color of the peel of the cv. Vlas fruit is due to substantial amounts of anthocyanins. Flavonoid biosynthesis genes showed a significantly lower expression level in the ripe fruit of the cv. Vlas in comparison with the cv. Snezhny. However, in both cultivars, transcripts of anthocyanin biosynthesis genes (DFR, ANS, UFGT) were not detected. Additionally, the purple fruit of the cv. Vlas accumulated more carotenoids and sucrose and less glucose and fructose than the white fruit of the cv. Snezhny. Biochemical data corresponded to the differential expression pattern of the key genes encoding the structural proteins of metabolism and transport of the compounds analyzed.

## Abstract (ru)

Баклажан (Solanum melongena L.) занимает пятое место по значимости среди овощных культур семейства Пасленовых, в том числе благодаря антиоксидантным свойствам плода за счет высокого содержания различных фенольных соединений. Наряду с популярными фиолетовоплодными сортами S. melongena имеются сорта, плоды которых синтезируют фенольные соединения, однако характеризуются белой окраской из-за отсутствия биосинтеза антоцианов. Определение количества антоцианов и других фенольных соединений, а также каротиноидов и сахаров входит в оценку качества плодов баклажана коммерческой (технической) спелости. Кроме антиоксидантных и вкусовых качеств, эти метаболиты связаны с устойчивостью плода к различным стрессовым факторам. В данном исследовании проведен сравнительный анализ содержания антоцианов, каротиноидов и растворимых сахаров (сахарозы, глюкозы, фруктозы) в кожице и мякоти плода как технической, так и биологической спелости у фиолетовоплодного (сорт Влас) и белоплодного (сорт Снежный) образцов баклажана отечественной селекции. Кожица и мякоть плода биологической спелости сортов Влас и Снежный были использованы для сравнительного транскриптомного анализа. Показано, что ключевые гены флавоноидного пути, метаболизма каротиноидов, гидролиза сахарозы, а также транспорта растворимых сахаров дифференциально экспрессируются между тканями плода как внутри каждого сорта, так и между сортами. Подтверждена связь фиолетовой окраски кожицы плода сорта Влас с присутствием значительных количеств антоцианов. Определено, что в сравнении с сортом Снежный спелый плод сорта Влас характеризуется существенно более низким уровнем экспрессии генов биосинтеза флавоноидов. Однако у обоих сортов в спелом плоде не выявлены транскрипты генов биосинтеза антоцианов (DFR, ANS, UFGT). Также показано, что в сравнении с белым плодом сорта Снежный фиолетовый плод сорта Влас накапливает больше каротиноидов и сахарозы и меньше глюкозы и фруктозы. Биохимические данные соответствуют профилю дифференциальной экспрессии ключевых генов, кодирующих структурные белки метаболизма и транспорта анализируемых соединений

## Introduction

Eggplant (Solanum melongena L.) is a vegetable crop that ranks fifth in economic importance in the nightshade family (Solanaceae). Despite its heat-loving nature, this crop is grown not only in tropical and subtropical climate zones, but also as a greenhouse crop in regions with cold climate (including the Russian Federation). The most famous are eggplant fruits with peel colored in different shades of purple, which is determined by the content of anthocyanins. The presence of anthocyanins and the fact that the fruit pulp is enriched with phenolic acids indicate powerful antioxidant properties of the eggplant fruit, classifying it as a product with high nutritional/dietary value (Gürbüz et al., 2018; Akhbari et al., 2019; Condurache et al., 2021; Saha et al., 2023).

In addition to purple-fruited varieties, there are also S. melongena varieties that produce fruits with white or green peel due to inhibition of anthocyanin biosynthesis (Condurache et al., 2021; Yang et al., 2022; You et al., 2022). The color (white, green, or intermediate shades) is determined by the ratio of two types of plastids in the cells of the fruit – chloroplasts and leucoplasts (Tao et al., 2023). For consumers, white-fruited varieties may be preferable because they lack the bitterness associated with dark-colored fruits due to changes in the content of glycoalkaloids (Lelario et al., 2019; Saha et al., 2023).

Commercial eggplant varieties are characterized by morphological variability, and screening of existing collections for a set of characteristics includes grouping by fruit peel color as the most important trait (Martínez-Ispizua et al., 2021). The assessment of fruit quality focuses on their antioxidant properties (including the determination of phenolic compounds/flavonoids, carotenoids and sugars), and there is wide variation in these terms (Martínez-Ispizua et al., 2021). Purple-fruited varieties, compared to white-fruited varieties, are characterized by greater antioxidant activity and increased content of phenols and carotenoids (both in the peel and in the pulp), and there is little or no difference in the total amount of sugars (Martínez-Ispizua et al., 2021; Colak et al., 2022).

There is no correlation between the content of flavonoids, carotenoids and sugars in eggplant fruits (Martínez-Ispizua et al., 2021). On the other hand, there is indirect evidence for the existence of such a phytohormone-mediated dependence in cherries (Teribia et al., 2016). Namely, there is an inverse correlation between the content of soluble sugars and transzeatin, as well as gibberellin GA4 and anthocyanins; in contrast, abscisic acid (ABA) is positively associated with the amount of anthocyanins and soluble sugars (Teribia et al., 2016). Moreover, the accumulation of anthocyanins is positively correlated with the amount of sugars in the Chinese date Ziziphus jujube (Jiang et al., 2020).

All of the antioxidant compounds mentioned, as well as soluble sugars, are closely related to resistance to various stress factors both in the vegetative part of the plant (Keunen et al., 2013; Pérez-Torres et al., 2021; Waadt et al., 2022) and in the fleshy fruit (Shi et al., 2019; Jiang et al., 2020). For example, it has been shown that increased production of phenolic compounds determines the resistance of the eggplant fruit to low temperatures (Shi et al., 2019). Elevated temperature has a positive effect on the content of sugars, anthocyanins, flavonoids and carotenoids in the fruits of the Chinese date Z. jujube, but in combination with drought it causes the opposite effect (Jiang et al., 2020).

This work aimed to characterize the fruits of two eggplant varieties, including the determination of the content of anthocyanins, carotenoids and soluble sugars, as well as the expression profile of key genes in the corresponding metabolic pathways. We chose two cultivars of domestic selection that have different fruit colors – white and purple, respectively. A significant difference from similar studies was that we analyzed fruits of not only technical (commercial) ripeness, but also those of biological ripeness

The following tasks were set: to obtain plant material (fruits of two varieties at the stages of technical and biological ripeness); to determine the content of target metabolites in the peel and pulp of fruits of technical and biological ripeness; to analyze transcriptomes of the peel and pulp of fruits at the stage of biological ripeness, focusing on transcripts of genes of target metabolic pathways; to validate transcriptomic data.

## Materials and methods

**Fig. 1.**
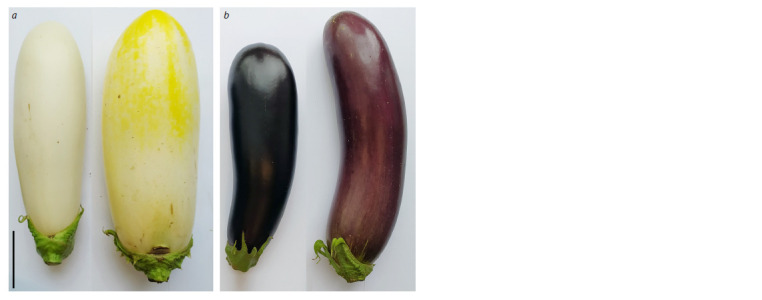
Photographs of the fruit of the eggplant cultivars Snezhny (a) and Vlas (b) in technical (CM; left) and biological (PR; right) ripeness. The cultivars differ in the color of the fruit peel – white (cv. Snezhny) and purple (cv. Vlas). Scale = 5 cm.

In a comparative study, we used accessions of two earlyripening eggplant varieties (S. melongena), originated by the Federal Scientific Vegetable Center (FSVC, Moscow region) that differed in the color of the ripe fruit. The fruits of cv. Snezhny (ID 9905014, https://gossortrf.ru/registry/) at the stage of technical ripeness have white peel and pulp. The fruits of cv. Vlas (ID 8057522) at technical ripeness have dark purple peel and white flesh. At the stage of biological ripeness, the fruit pulp remains white in both cultivars, and the peel acquires yellowish (cv. Snezhny) or brown (cv. Vlas) shades (Fig. 1).

Plants of the studied varieties were grown (2023) until the fruiting stage in a film greenhouse of the Federal Research Vegetable Center. In August, fruits were collected at technical (commercially mature, CM) and biological (physiologically ripe, PR) ripeness, separated into peel (exocarp) and pulp (mesocarp), grinded in a porcelain mortar in liquid nitrogen and used for biochemical, metabolomic and transcriptomic analyses.

The content of anthocyanins and carotenoids was determined spectrophotometrically in chloroform-methanol extracts according to (Filyushin et al., 2020). Since delphinidin glycosides (93–98 % of the total) dominate among the anthocyanins accumulated in the peel of eggplant fruit (Condurache et al., 2021; Yang et al., 2022), the anthocyanin content was calculated in terms of delphinidin-3-rutinoside.

The content of sugars (glucose, fructose and sucrose) was determined according to metabolome data (unpublished), which were obtained according to (Filyushin et al., 2023a). In short, approximately 0.2 g of finely ground tissue was extracted twice with 200 μl of 80 % methanol. The total extract was evaporated, dissolved in 30 % methanol (at the rate of 50 mg wet weight per 100 μl of the extract) and subjected to mass spectral analysis using ultra-performance liquid chromatography-quadrupole time-of-flight mass spectrometry (UPLC-qTOF-MS/MS) according to the protocol [https://lcms.cz/labrulez-bucket-strapi-h3hsga3/1866243_ lcms_148_how_potato_fights_its_enemies_02_2019_ebook_ rev_01_9d3990d6c4/1866243-lcms-148-how-potatofights- its-enemies-02-2019-ebook-rev-01.pdf]. The signal level/100 mg of annotated compounds was used as a relative indicator for sugar content.

Differentially expressed genes (DEGs) encoding proteins involved in sucrose hydrolysis and transport of soluble sugars (invertases and sugar uniporters) were determined from transcriptome data for the peel and pulp of the PR fruit (unpublished). For transcriptomic analysis, preparations of total RNA were isolated (RNeasy Plant Mini Kit, Qiagen, USA) and used for mRNA libraries (NEBNext® mRNA Library Prep Reagent Set for Illumina; New England BioLabs, USA), which were then sequenced (Illumina HiSeq2500; Illumina Inc., USA). Trinity v3.5.13 (https://github.com/trinityrnaseq/ trinityrnaseq/wiki) and TransDecoder v5.1.0 (https://github. com/TransDecoder/TransDecoder) were used to assemble and determine coding sequences; CDSs were annotated using NCBI-Blast (https://www.ncbi.nlm.nih.gov/). Relative transcript levels (FPKM; number of fragments per kb transcripts per million mapped fragments) were estimated using RSEM (https://github.com/deweylab/RSEM). To determine DEGs both within varieties (peel vs. pulp) and between varieties (peel vs. peel; pulp vs. pulp), transcriptome data were normalized to the transcript number of the reference gene GAPDH.

Structural analysis of DEGs was performed using NCBIBLAST (https://blast.ncbi.nlm.nih.gov/Blast.cgi) and MEGA 7.0 (https://www.megasoftware.net/) using genomic (GCA_000787875.1) (Hirakawa et al., 2014) and transcriptomic (https://www.ncbi.nlm.nih.gov/) S. melongena data.

Transcriptomic data were validated using quantitative realtime PCR (qRT-PCR) and a CFX96 Real-Time PCR Detection System (Bio-Rad Laboratories, USA); qRT-PCR program [95 °C – 5 min.; 40 cycles (95 °C – 15 s, 62 °C – 50 s)]. Based on available total RNA preparations, cDNA was synthesized (GoScript™ Reverse Transcription System, Promega, USA) and 3 ng was used in the reaction. The reaction mixture included the “Reaction mixture for qRT-PCR in the presence of SYBR GreenI and ROX” (Sintol LLC, Russia) and genespecific primers. Reactions were performed in three technical and two biological replicates and normalized to the transcript level of the reference gene GAPDH (Zhang et al., 2014).

The obtained biochemical and expression data were statistically processed in GraphPad Prism v.8 (GraphPad Software Inc., USA; https://www.graphpad.com/scientific-software/ prism/). To assess the significance of the differences, a t-test was used ( p < 0.05 indicates statistical significance of the differences).

## Results

The study was focused on the comparative characteristics of the fruit (technical and biological ripeness) of two eggplant varieties belonging to the same species, S. melongena, and differing in the color of the fruit peel. Namely, cv. Snezhny and cv. Vlas with white/yellowish and purple/brown, respectively, colors of the fruit peel at technical/biological ripeness (Fig. 1).

**Fig. 2.**
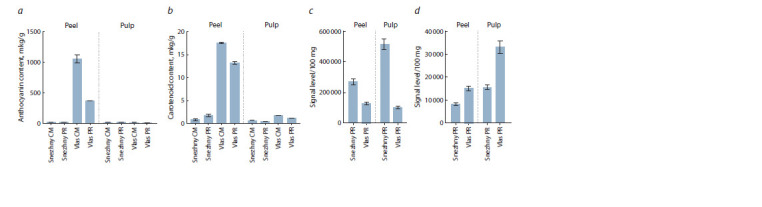
The content of the sum of anthocyanins (a), the sum of carotenoids (b), hexoses (total glucose and fructose) (c) and sucrose (d) in the peel and pulp of the fruit of technical (CM) and biological (PR) ripeness of eggplant cultivars Snezhny and Vlas (S. melongena). The signal level/100 mg of annotated compounds was used as a relative indicator for sugar content obtained from non-targeted metabolomic profiling.

A biochemical analysis of the peel and pulp in the dynamics of fruit ripening showed that the content of anthocyanins corresponds to the color of the analyzed fruit tissues of biological ripeness. In the yellowish peel and white pulp of the fruit of cv. Snezhny, as well as the white pulp of the fruit of cv. Vlas, the amount of anthocyanins showed trace values, while in the purple-brown peel of the fruit of cv. Vlas anthocyanins amount was ~300 times higher (Fig. 2a).

The fruits of both varieties, both in technical and biological ripeness, contained traces of carotenoids in the pulp. In the peel, carotenoids accumulated more actively: in cv. Vlas, the amount of carotenoids was ~25 times higher than in cv. Snezhny (Fig. 2b).

If the differences in the content of anthocyanins in the analyzed varieties were predictable, then the significant differences in the content of soluble sugars were somewhat unexpected. According to metabolomic profiling of the peel and pulp, it was found that the fruit of cv. Snezhny contains ~2 (peel) and ~5 (pulp) times more hexoses (glucose, fructose), as well as ~2 (peel and pulp) times less sucrose than the fruit of cv. Vlas (Fig. 2c).

A comparative analysis of the transcriptomes of the peel and pulp of the fruit of the Snezhny and Vlas varieties identified a number of DEGs, which, as expected (according to the results of biochemical and metabolomic analyses), included genes associated with the metabolism of anthocyanins, carotenoids and sugars (see the Table).

**Table 1.**
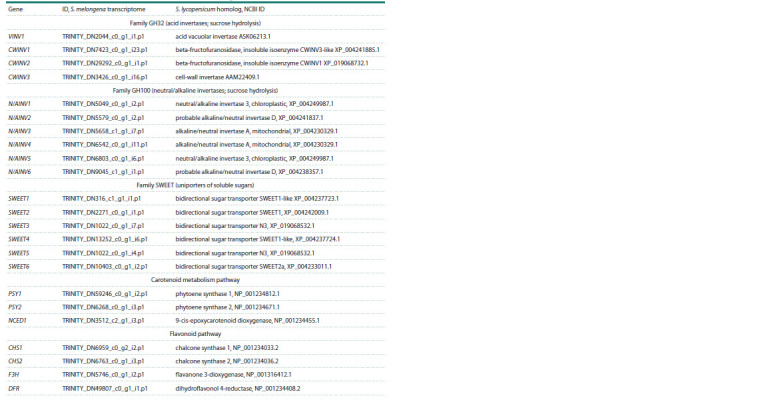
List of DEGs associated with the metabolism of sugars, carotenoids and anthocyanins

**Fig. 3.**
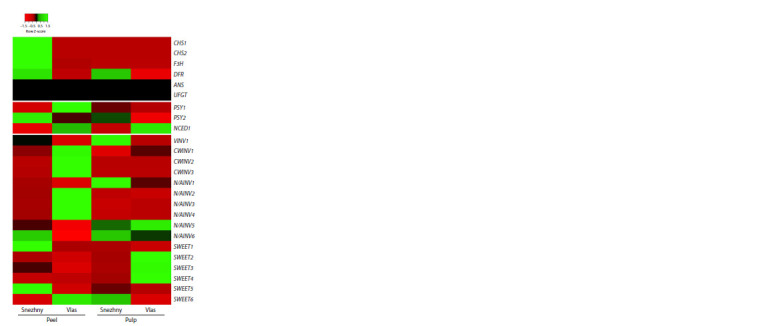
Heatmap of the expression of DEGs associated with the metabolism of anthocyanins and carotenoids, as well as with the hydrolysis of sucrose and the transport of soluble sugars in the peel and pulp of biologically ripe fruit (PR) of the Snezhny and Vlas cultivars (S. melongena). The heatmap was constructed based on transcriptomic analysis data.

It was found that the key genes of the flavonoid pathway (Zhang et al., 2014; Alappat B., Alappat J., 2020) before anthocyanin synthesis (CHS1, CHS2, F3H) are highly transcribed in the peel of the fruit of cv. Snezhny and are detected in significantly smaller and similar quantities in the pulp (both varieties) and peel (cv. Vlas) (Fig. 3). Considering the branch of the pathway related to anthocyanin synthesis, the level of expression of the first gene of the branch, DFR, in the peel and pulp of the fruit of cv. Snezhny is significantly higher than that of cv. Vlas. However, the number of transcripts in the FPKM value for DFR is extremely low in all four samples (0.49–3.44), so we cannot speak of a significant difference between varieties, since the level of gene transcripts approaches zero. At the same time, transcripts of subsequent genes of the anthocyanin biosynthesis branch – ANS (anthocyanidin synthase) and UFGT (UDP-glucosoflavonoid-3-Oglucosyltransferase) – were not included in the list of DEGs and were detected in trace amounts (Fig. 3).

Analysis of transcripts of phytoene synthase genes, key isoenzymes of carotenoid metabolism (Rosas-Saavedra, Stange, 2016), showed trace PSY1 values in the peel (cv. Snezhny) and pulp (both varieties) of the fruit and significant PSY1 expression level in the fruit peel of cv. Vlas (Fig. 3). Relatively significant numbers of PSY2 transcripts were found in the fruit peel (both varieties) and pulp (cv. Snezhny). At the same time, the number of PSY2 transcripts was significantly higher in cv. Snezhny compared to cv. Vlas (Fig. 3). Another DEG associated with carotenoid catabolism, the 9-cis-epoxycarotenoid dioxygenase gene (NCED1), which catalyzes the synthesis of ABA from xanthophylls of the β,β-branch of the pathway (Rosas-Saavedra, Stange, 2016), was highly transcribed in the fruit peel and pulp of cv. Vlas, while in the fruit of cv. Snezhny, only trace values were detected (Fig. 3)

The list of DEGs associated with irreversible hydrolysis of sucrose and transport of mono- and disaccharides included genes for vacuolar invertase (VINV1), cell wall invertases (CWINV1–3), neutral/alkaline invertases (N/AINV1–6) and sugar uniporters (SWEET1–6) (see the Table).

In the peel of the fruit of cv. Snezhny, the highest level of expression was observed for the genes of four invertases (VINV1, CWINV1, N/AINV5 and 6) and three sugar uniporters (SWEET1, 3 and 5); in the fruit pulp – for the genes of four invertases (VINV1, N/AINV1, 5 and 6) and two sugar uniporters (SWEET5 and 6) (Fig. 3).

In general, cv. Vlas differed from cv. Snezhny in higher expression levels and a larger number of DEGs for invertases and sugar uniporters. In the fruit peel of cv. Vlas, the genes of six invertases (CWINV3, CWINV1 and 2, N/AINV2–4) and one sugar uniporter (SWEET6) were most highly transcribed, while in the fruit pulp, four invertases (CWINV1, N/AINV1, 5 and 6) and three sugar uniporters (SWEET2–4) (Fig. 3).

Thus, the expression profile of genes for the metabolism of anthocyanins, carotenoids and sugars varied both within each cultivar (peel vs. pulp) and between cultivars (peel vs. peel, pulp vs. pulp).

**Fig. 4.**
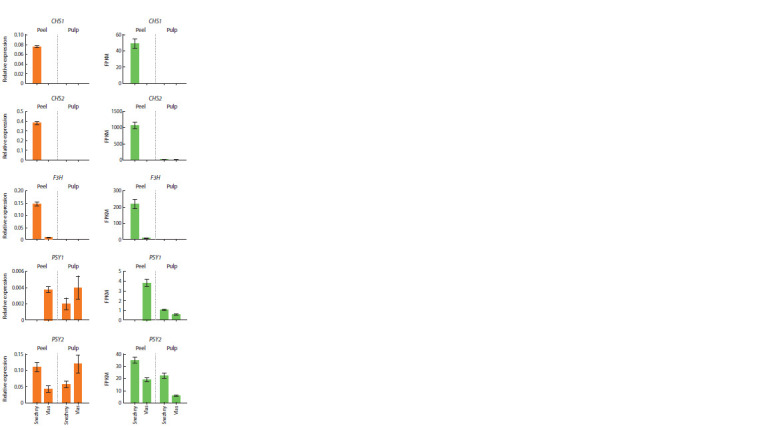
Relative expression of the CHS1, CHS2, F3H, PSY1 and PSY2 genes based on qRT-PCR (left) and transcriptome (right) data. The absence of the DFR and ANS transcripts was also confirmed by qRT-PCR; graphs are not shown. The primer sequences for CHS1, CHS2, F3H, DFR, ANS and the reference gene GAPDH were taken from the paper (Filyushin et al., 2023b); for the PSY1 and PSY2 genes, from (Kulakova et al., 2023).

Transcriptomic data were validated using qRT-PCR. Namely, in the same fruit tissues, the expression of CHS1, CHS2, F3H, DFR, ANS (flavonoid pathway), PSY1 and PSY2 (carotenogenesis) was determined (Fig. 4). It was shown that the expression pattern of these genes is consistent with transcriptomic data, with the exception of insignificant differences in the ratio of expression levels of the PSY1 and PSY2 genes in the fruit pulp between varieties (Fig. 4).

## Discussion

The morphological diversity of eggplant cultivars has been the subject of much research, facilitating the optimization of breeding new cultivars with improved characteristics (Martínez-Ispizua et al., 2021). Particular attention is paid to metabolites (content, regulation of synthesis/accumulation) that have antioxidant properties and/or determine the ontogeny/ stress resistance and taste of the fruit (Martínez-Ispizua et al., 2021). Nutraceuticals considered mainly include polyphenols, ascorbic acid, carotenoids and, less commonly, glycoalkaloids and sugars (Gürbüz et al., 2018; Akhbari et al., 2019; Condurache et al., 2021; Martínez-Ispizua et al., 2021; Saha et al., 2023)

In this study, accessions of two eggplant S. melongena varieties were characterized, which differ in the color of the fruit peel: cv. Snezhny (white color) and cv. Vlas (purple color) (Fig. 1). The characterization included the content of the sum of anthocyanins, the sum of carotenoids and soluble sugars in the peel and pulp of the fruit (CM and PR), accompanied by an analysis of the expression of genes encoding the key stages of metabolism of these compounds in the tissues of the biologically ripe fruit (PR).

Biochemical analysis confirmed that the purple color of the fruit peel of cv. Vlas is due to the presence of anthocyanins (Fig. 2a). The significantly higher content of carotenoids in the fruit peel of cv. Vlas in comparison with the pulp, as well as with the fruit of cv. Snezhny (Fig. 2b), does not affect the color of the fruit, apparently due to the presence of a large amount of anthocyanins

In the fruit of cv. Vlas, the content of both pigments decreased significantly during the transition from technical to biological ripeness (Fig. 2a, b). This may be due to a decrease in the expression of genes for the biosynthesis of these metabolites or to the accelerated catabolism of pigment compounds. A decrease in concentration was also observed for soluble sugars (Fig. 2c, d). These results correspond to a decrease in the taste and antioxidant characteristics of the fruit at the stage of biological ripeness and explain the commercial use of fruits of technical ripeness.

According to transcriptomic analysis, genes for the metabolism of anthocyanins, carotenoids and sugars are differentially expressed both between fruit tissues within the same variety and between varieties (see the Table). This presumably determines intra- and intervarietal differences in the content of the corresponding compounds in fruit tissues

In general, the obtained data on the expression of flavonoid pathway genes correspond to the previously shown profile of their expression in eggplant varieties with white and purple peel (Filyushin et al., 2023b). According to these data, between the stages of technical and biological ripeness, significant changes occur in the expression of genes of the flavonoid pathway, resulting in decrease of the content of anthocyanins in the peel of the purple fruit

An unexpected result was the significantly higher expression of key genes of the flavonoid pathway (up to the anthocyanin branch) in the fruit of cv. Snezhny in comparison with the fruit of cv. Vlas (Fig. 3), which indicates the possibility of more flavonoids (excluding anthocyanins) being synthesized in the fruit of cv. Snezhny. Since the content of carotenoids in the fruit of cv. Snezhny is minimal, and the expression of flavonoid pathway genes is relatively high, it can be assumed that the yellow color of the ripe fruit (PR) of cv. Snezhny (Fig. 1a) is associated with the accumulation of flavonoids (colorless or yellow in color). This distinguishes eggplant fruits from the fruits of related species, tomato (S. lycopersicum) and pepper (Capsicum annuum), the color of which is associated with the accumulation of carotenoids (Filyushin et al., 2020).

In addition, these results are contrary to the few studies comparing the content of phenolic compounds in white and purple eggplant fruits, which indicate a greater accumulation of phenolic compounds in purple fruits (Martínez-Ispizua et al., 2021; Colak et al., 2022). Both studies included the analysis of only one white-fruited variety (Martínez-Ispizua et al., 2021; Colak et al., 2022), as in our case. Thus, whitefruited eggplant varieties can differ significantly from each other in the content of phenolic compounds and, consequently, antioxidant activity.

The shown expression profile of the phytoene synthase genes (PSY1, PSY2) initiating the biosynthesis of carotenoids (Fig. 3, 4) corresponds to the specificity of each of the two isoenzymes to a certain type of plastid (Rosas-Saavedra, Stange, 2016). Thus, PSY1, encoding a chromoplast-specific enzyme, was expressed in trace amounts, while chloroplast-specific PSY2 corresponded to an order of magnitude more transcripts (Fig. 3, 4). At the same time, a high level of expression of the 9-cis-epoxycarotenoid dioxygenase (NCED1) gene, which catalyzes the conversion of β,β-branch carotenoids into ABA (Rosas-Saavedra, Stange, 2016), in the fruit of cv. Vlas, and its trace amounts in the fruit of cv. Snezhny (Fig. 3) suggest increased ABA content in the purple-colored fruit. Taking into account the complex functions of ABA (Waadt et al., 2022), this fact may indicate a greater efficiency of development, ripening, and response to stress factors of the purple fruit compared to the white fruit.

ABA content is positively associated with the amount of anthocyanins and soluble sugars (Teribia et al., 2016), although the content of the latter does not correlate with the accumulation of phenolic compounds, as well as carotenoids (Martínez-Ispizua et al., 2021).

The concentration of soluble sugars is regulated, among other things, by hydrolysis (invertases) and transport between tissues (sugar transporters) (Liu et al., 2022; Ren et al., 2022; Filyushin et al., 2023c). The invertase family includes neutral/ alkaline (N/AINV) and acidic (vacuolar and cell wall; VINV/ CWINV) enzymes that are involved in the regulation of plant ontogeny and stress tolerance (Qian et al., 2016), as well as sugar uniporters of the SWEET family (Fan et al., 2023; Filyushin et al., 2023a).

In comparison with cv. Vlas, the fruits of cv. Snezhny contained more hexoses and less sucrose (Fig. 2), which, at first glance, contradicts the lower activity of invertase genes (Fig. 3). However, these discrepancies may be a consequence of incomplete correspondence of the fruits of the two analyzed varieties in terms of the degree of biological ripeness. Ripe, fleshy fruits are characterized by enlarged cells with large vacuoles that actively accumulate and store sugars (Hedrich et al., 2015). In the peel and pulp of the fruit of cv. Snezhny, the highest level of expression of the only found DEG of vacuolar invertase, VINV1, is detected (Fig. 3), which corresponds to the highest content of hexoses there (Fig. 2) and is probably a sign of complete biological ripeness of the analyzed fruit. At the same time, in the fruit of cv. Vlas, cell wall invertases and neutral/alkaline invertases are highly expressed (Fig. 3), operating in the cytoplasm and chloroplasts (Qian et al., 2016), where hexoses are actively utilized for development processes (Hedrich et al., 2015). That is, the analyzed fruit of a given variety may not have yet reached full ripening and is at an intermediate stage preceding biological ripeness. Also, the observed intervarietal difference in the content of sugars in the fruit may be a consequence of transport regulation of their concentration, including with the help of uniporters of the SWEET family (Filyushin et al., 2023a).

## Conclusion

Thus, in this study, a comparative characterization of the ripe fruit of two varieties of eggplant S. melongena with white (cv. Snezhny) and purple (cv. Vlas) peel color was carried out using biochemical and transcriptomic analyses. It was shown that the purple color of the fruit of cv. Vlas is associated with the presence of anthocyanins and is accompanied by an increased accumulation of carotenoids and sucrose. This is consistent with the expression profile of genes linked to the key stages of the metabolism of these compounds and the transport of soluble sugars. Compared to cv. Vlas, the fruit of cv. Snezhny is characterized by a large content of hexoses and, possibly, flavonoids.

## Conflict of interest

The authors declare no conflict of interest.

## References

Akhbari M., Hamedi S., Aghamiri Z.S. Optimization of total phenol and
anthocyanin extraction from the peels of eggplant (Solanum melongena
L.) and biological activity of the extracts. J. Food Measure.
Character. 2019;13:3183-3197. DOI 10.1007/s11694-019-00241-1

Alappat B., Alappat J. Anthocyanin pigments: beyond aesthetics. Molecules.
2020;25(23):5500. DOI 10.3390/molecules25235500

Colak N., Kurt-Celebi A., Gruz J., Strnad M., Hayirlioglu-Ayaz S.,
Choung M.G., Esatbeyoglu T., Ayaz F.A. The phenolics and antioxidant
properties of black and purple versus white eggplant cultivars.
Molecules. 2022;27(8):2410. DOI 10.3390/molecules27082410

Condurache N.N., Croitoru C., Enachi E., Bahrim G.E., Stanciuc N.,
Rapeanu G. Eggplant peels as a valuable source of anthocyanins:
extraction, thermal stability and biological activities. Plants. 2021;
10:577. DOI 10.3390/Plants10030577

Fan X.W., Sun J.L., Cai Z., Zhang F., Li Y.Z., Palta J.A. MeSWEET15a/b
genes play a role in the resistance of cassava (Manihot esculenta
Crantz) to water and salt stress by modulating sugar distribution.
Plant Physiol. Biochem. 2023;194:394-405. DOI 10.1016/j.plaphy.
2022.11.027

Filyushin M.A., Dzhos E.A., Shchennikova A.V., Kochieva E.Z. Dependence
of pepper fruit colour on basic pigments ratio and expression
pattern of carotenoid and anthocyanin biosynthesis genes.
Russ. J. Plant Physiol. 2020;67(6):1054-1062. DOI 10.1134/S1021
443720050040

Filyushin M.A., Anisimova O.K., Shchennikova A.V., Kochieva E.Z.
Genome-wide identification, expression, and response to Fusarium
infection of the SWEET gene family in garlic (Allium sativum L.).
Int. J. Mol. Sci. 2023a;24(8):7533. DOI 10.3390/ijms24087533

Filyushin M.A., Shchennikova A.V., Kochieva E.Z. Coexpression of
structural and regulatory genes of the flavonoid pathway reveals
the characteristics of anthocyanin biosynthesis in eggplant organs
(Solanum melongena L.). Russ. J. Plant Physiol. 2023b;70:27. DOI
10.1134/S1021443722603147

Filyushin M.A., Slugina M.A., Shchennikova A.V., Kochieva E.Z. Differential
expression of sugar uniporter genes of the SWEET family
in the regulation of qualitative fruit traits in tomato species (Solanum
section Lycopersicon). Russ. J. Plant Physiol. 2023c; 70(4):70. DOI
10.1134/S102144372360023X

Gürbüz N., Uluişikb S., Frarya A., Fraryc A., Doğanlara S. Health
benefits
and bioactive compounds of eggplant. Food Chem. 2018;
268:602. DOI 10.1016/j.foodchem.2018.06.093

Hedrich R., Sauer N., Neuhaus H.E. Sugar transport across the plant
vacuolar membrane: nature and regulation of carrier proteins. Curr.
Opin. Plant Biol. 2015;25:63-70. DOI 10.1016/j.pbi.2015.04.008

Hirakawa H., Shirasawa K., Miyatake K., Nunome T., Negoro S.,
Ohyama A., Yamaguchi H., Sato S., Isobe S., Tabata S., Fukuoka H.
Draft genome sequence of eggplant (Solanum melongena L.): the
representative solanum species indigenous to the old world. DNA
Res. 2014;21:649. DOI 10.1093/dnares/dsu027

Jiang W., Li N., Zhang D., Meinhardt L., Cao B., Li Y., Song L. Elevated
temperature and drought stress significantly affect fruit quality and
activity of anthocyanin-related enzymes in jujube (Ziziphus jujuba
Mill. cv. ‘Lingwuchangzao’). PLoS One. 2020;15(11):e0241491.
DOI 10.1371/journal.pone.0241491

Keunen E., Peshev D., Vangronsveld J., Van Den Ende W., Cuypers A.
Plant sugars are crucial players in the oxidative challenge during
abiotic stress: extending the traditional concept. Plant Cell Environ.
2013;36(7):1242-1255. DOI 10.1111/pce.12061

Kulakova A.V., Shchennikova A.V., Kochieva E.Z. Expression of carotenoid
biosynthesis genes during the long-term cold storage of
potato tubers. Russ. J. Genet. 2023;59(8):794-807. DOI 10.1134/
S1022795423080094

Lelario F., De Maria S., Rivelli A.R., Russo D., Milella L., Bufo S.A.,
Scrano L. A complete survey of glycoalkaloids using LC-FTICRMS
and IRMPD in a commercial variety and a local landrace of
eggplant (Solanum melongena L.) and their anticholinesterase and
antioxidant activities. Toxins (Basel). 2019;11(4):230. DOI 10.3390/
toxins11040230

Liu Y.H., Song Y.H., Ruan Y.L. Sugar conundrum in plant-pathogen
interactions: roles of invertase and sugar transporters depend on pathosystems.
J. Exp. Bot. 2022;73(7):1910-1925. DOI 10.1093/jxb/
erab562

Martínez-Ispizua E., Calatayud Á., Marsal J.I., Mateos-Fernández R.,
Díez M.J., Soler S., Valcárcel J.V., Martínez-Cuenca M.R. Phenotyping
local eggplant varieties: commitment to biodiversity and
nutritional quality preservation. Front. Plant Sci. 2021;12:696272.
DOI 10.3389/fpls.2021.696272

Pérez-Torres I., Castrejón-Téllez V., Soto M.E., Rubio-Ruiz M.E.,
Manzano-Pech L., Guarner-Lans V. Oxidative stress, plant natural
antioxidants, and obesity. Int. J. Mol. Sci. 2021;22(4):1786. DOI
10.3390/ijms22041786

Qian W., Yue C., Wang Y., Cao H., Li N., Wang L., Hao X., Wang X.,
Xiao B., Yang Y. Identification of the invertase gene family (INVs)
in tea plant and their expression analysis under abiotic stress.
Plant Cell Rep. 2016;35(11):2269-2283. DOI 10.1007/s00299-016-
2033-8

Ren R., Wan Z., Chen H., Zhang Z. The effect of inter-varietal variation
in sugar hydrolysis and transport on sugar content and photosynthesis
in Vitis vinifera L. leaves. Plant Physiol. Biochem. 2022;189:
1-13. DOI 10.1016/j.plaphy.2022.07.031

Rosas-Saavedra C., Stange C. Biosynthesis of carotenoids in plants:
enzymes and color. Subcell. Biochem. 2016;79:35-69. DOI 10.1007/
978-3-319-39126-7_2

Saha P., Singh J., Bhanushree N., Harisha S.M., Tomar B.S., Rathinasabapathi
B. Eggplant (Solanum melongena L.) nutritional and
health promoting phytochemicals. In: Kole C. (Ed.). Compendium
of Crop Genome Designing for Nutraceuticals. Singapore: Springer
Nature Singapore, 2023;1463-1493. DOI 10.1007/978-981-19-
4169-6_53

Shi J., Zuo J., Xu D., Gao L., Wang Q. Effect of low-temperature conditioning
combined with methyl jasmonate treatment on the chilling resistance of eggplant (Solanum melongena L.) fruit. J. Food
Sci. Technol. 2019;56(10):4658-4666. DOI 10.1007/s13197-019-
03917-0

Tao T., Hu W., Yang Y., Zou M., Zhou S., Tian S., Wang Y. Transcriptomics
reveals the molecular mechanisms of flesh colour differences
in eggplant (Solanum melongena). BMC Plant Biol. 2023;23(1):5.
DOI 10.1186/s12870-022-04002-z

Teribia N., Tijero V., Munné-Bosch S. Linking hormonal profiles with
variations in sugar and anthocyanin contents during the natural development
and ripening of sweet cherries. Nat. Biotechnol. 2016;
33(6):824-833. DOI 10.1016/j.nbt.2016.07.015

Waadt R., Seller C.A., Hsu P.K., Takahashi Y., Munemasa S., Schroeder
J.I. Plant hormone regulation of abiotic stress responses. Nat.
Rev. Mol. Cell Biol. 2022;23(10):680-694. DOI 10.1038/s41580-
022-00479-6

Yang G., Li L., Wei M., Li J., Yang F. SmMYB113 is a key transcription
factor responsible for compositional variation of anthocyanin
and color diversity among eggplant peels. Front. Plant Sci. 2022;13:
843996. DOI 10.3389/fpls.2022.843996

You Q., Li H., Wu J., Li T., Wang Y., Sun G., Li Z., Sun B. Mapping
and validation of the epistatic D and P genes controlling anthocyanin
biosynthesis in the peel of eggplant (Solanum melongena L.) fruit.
Hortic. Res. 2022;10(2):uhac268. DOI 10.1093/hr/uhac268

Zhang Y., Hu Z., Chu G., Huang C., Tian S., Zhao Z., Chen G. Anthocyanin
accumulation and molecular analysis of anthocyanin biosynthesis-
associated genes in eggplant (Solanum melongena L.).
J. Agric.
Food Chem. 2014;62:2906. DOI 10.1021/jf404574c

